# Dose Effect and Mode of Inheritance of Diabetogenic Gene on Mouse Chromosome 11

**DOI:** 10.1155/2013/608923

**Published:** 2013-02-25

**Authors:** Naru Babaya, Hironori Ueda, Shinsuke Noso, Yoshihisa Hiromine, Koji Nojima, Michiko Itoi-Babaya, Misato Kobayashi, Tomomi Fujisawa, Hiroshi Ikegami

**Affiliations:** ^1^Department of Endocrinology, Metabolism and Diabetes, Kinki University Faculty of Medicine, 377-2 Ohno-higashi, Osaka-sayama, Osaka 589-8511, Japan; ^2^Department of Molecular Endocrinology, Osaka University Graduate School of Medicine, Suita, Osaka 565-0871, Japan; ^3^Department of Geriatric Medicine, Osaka University Graduate School of Medicine, Suita, Osaka 565-0871, Japan; ^4^Department of Applied Molecular Bioscience, Graduate School of Bioagricultural Sciences, Nagoya University, Nagoya, Aichi 464-8601, Japan

## Abstract

The quantitative trait locus (QTL) mapping in segregating crosses of NSY (Nagoya-Shibata-Yasuda) mice, an animal model of type 2 diabetes, with nondiabetic strain C3H/He mice has identified diabetogenic QTLs on multiple chromosomes. The QTL on chromosome 11 (Chr11) (*Nidd1n*) showing the largest effect on hyperglycemia was confirmed by our previous studies with homozygous consomic mice, C3H-11^NSY^, in which the NSY-derived whole Chr11 was introgressed onto control C3H background genes. C3H-11^NSY^ mice also showed a streptozotocin (STZ) sensitivity. In the present study, we constructed heterozygous C3H-11^NSY^ mice and the phenotypes were analyzed in detail in comparison with those of homozygous C3H-11^NSY^ and C3H mice. Heterozygous C3H-11^NSY^ mice had significantly higher blood glucose levels and STZ sensitivity than those in C3H mice. Hyperglycemia and STZ sensitivity in heterozygous C3H-11^NSY^ mice, however, were not as severe as in homozygous C3H-11^NSY^ mice. The body weight and fat pad weight in heterozygous C3H-11^NSY^ mice were similar to those in C3H and homozygous C3H-11^NSY^ mice. These data indicated that the introgression of Chr11 of the diabetes-susceptible NSY strain onto diabetes-resistant C3H caused marked changes in the glucose tolerance and STZ susceptibility even in a heterozygous state, and suggested that the mode of inheritance of a gene or genes on Chr11 for hyperglycemia and STZ sensitivity is additive.

## 1. Introduction

Patients with type 2 diabetes have a complex phenotype with impaired insulin secretion, insulin resistance both in the liver and peripheral tissues, and increased hepatic glucose production, all of which contribute to the development of overt hyperglycemia [1]. Genetic dissection of type 2 diabetes in humans is difficult because of the genetic heterogeneity, multigenicity, and environmental variation. One way to overcome this complexity in the human population is to use well-defined animal models of type 2 diabetes.

The NSY (Nagoya-Shibata-Yasuda) mouse is an inbred animal model with a spontaneous development of type 2 diabetes [2, 3]. The animal is moderately obese, and both impaired insulin response to glucose and insulin resistance contribute to diabetes [2, 3]. Quantitative trait locus (QTL) mapping in segregating crosses of NSY with the nondiabetic strain C3H/He has identified diabetes-related QTLs on multiple chromosomes [4, 5]. Using the consomic strategy, we previously dissected a quantitative trait locus (QTL), *Nidd1n*, on chromosome 11 (Chr11) affecting glucose-related phenotypes [6, 7]. The constructed strain, C3H-11^NSY^, which carries the homozygous NSY-derived diabetes-susceptible Chr11 on C3H-derived diabetes-resistant background genes, showed hyperglycemia [6] and streptozotocin (STZ) sensitivity [7] compared with control C3H strain. Although these data clearly demonstrated the presence of the diabetogenic gene(s) on mouse Chr11, the dose effect and the mode of inheritance of the gene(s) responsible for each diabetes-related phenotype are still unclear. In the present study, we constructed heterozygous C3H-11^NSY^ mice, and the phenotypes were analyzed in detail in comparison with those of homozygous C3H-11^NSY^ and C3H mice.

## 2. Materials and Methods

### 2.1. Animals

NSY mice [2] were originally obtained from the Branch Hospital of the Nagoya University School of Medicine. C3H/He mice were purchased from Charles River Laboratories (Kanagawa, Japan). These strains were maintained by a brother-sister mating. Maintained male C3H/He mice were used in this study (*n* = 8 for Study 1, *n* = 20 for Study 2).

The consomic strain, C3H-11^NSY^, was previously constructed using a marker-assisted method [6, 7]. Shortly, F1 male mice were obtained by mating (NSY × C3H/He). These males were then mated with C3H/He females, and their male progeny, heterozygous for Chr11, were used for the next generation. This process was repeated until all the markers for background typing became homozygous for the C3H genotype (N6 or N7), at which point a heterozygous consomic strain was obtained and the male mice were used in this study (heterozygous C3H-11^NSY^: *n* = 8 for Study 1).

Mice heterozygous for Chr11 were intercrossed to obtain mice homozygous for Chr11. Constructed homozygous C3H-11^NSY^ mice were maintained by brother-sister mating. To analyze male heterozygous C3H-11^NSY^ mice in this study (*n* = 20 for Study 2), male homozygous C3H-11^NSY^ were mated with female C3H/He mice.

All mice had free access to tap water and a standard diet (CRF-1: Oriental Yeast, Tokyo, Japan) in an air-conditioned room (22–25°C) with a 12 h light-dark cycle (6 : 00–18 : 00 h) in the animal facilities of the Osaka University Graduate School of Medicine. The experiment was approved by the Osaka University Graduate School of Medicine Ethics Committee. 

### 2.2. Analysis of Hyperglycemia-Related Phenotypes in Heterozygous C3H-11^NSY^ and C3H (Study 1)

Glucose tolerance was assessed by intraperitoneal glucose tolerance test (ipGTT) (2 g glucose/kg body weight) in overnight-fasted mice at 48 weeks of age, and blood glucose levels were measured at 0, 30, 60, 90, and 120 min using Glutest E (Kyoto Daiichi Kagaku, Kyoto, Japan). The area under the glucose curve (gAUC) was calculated according to the trapezoid rule from the glucose measurements at 0, 30, 60, 90, and 120 min.

Insulin secretion in response to glucose was assessed by intraperitoneal glucose tolerance test (ipGTT) (2 g glucose/kg body weight) in overnight-fasted mice at 52 weeks of age, and blood glucose levels and plasma insulin levels were measured at 0, 15, and 30 min. Plasma insulin level was measured using an enzyme-immunosorbent assay (ELISA) kit (Morinaga, Yokohama, Japan). Insulin values in micrograms per liter obtained by ELISA were converted to picomoles per liter by multiplying by a factor of 174. Incremental glucose (ΣΔ glucose) and incremental insulin (ΣΔ insulin) were calculated from the measurements at 0, 15, and 30 min. Insulinogenic index was calculated as ΣΔ insulin divided by ΣΔ glucose. Insulin resistance was assessed by HOMA-IR, which was calculated from the basal insulin and glucose concentrations.

Anatomical analysis was performed at 54 weeks of age. After body weight measurement, mice were killed under sevoflurane. The liver, epididymal fat pads, and mesenteric fat pads were dissected and weighed.

Hyperglycemia-related phenotypes were compared between heterozygous C3H-11^NSY^ and C3H and between heterozygous C3H-11^NSY^ and homozygous C3H-11^NSY^, which have been reported previously [6].

### 2.3. Analysis of Streptozotocin Sensitivity in Heterozygous C3H-11^NSY^ (Study 2)

The mice received a single injection of STZ at a dose of 175 mg/kg body weight at 12 weeks of age. STZ was dissolved in sodium citrate buffer (Wako Pure Chemical Industries, Ltd., Osaka, JAPAN) and immediately injected intraperitoneally. Blood glucose level and body weight were measured on days 0, 1, 2, 4, 5, 7, and 8 after injection. Mice with a blood glucose level higher than 11.1 mmol/L were considered hyperglycemic. Homozygous C3H-11^NSY^ and C3H data have been reported previously [7] and reanalyzed in this study. Although data of a small number of heterozygous C3H-11^NSY^ have been described [7], we constructed a large number of heterozygous C3H-11^NSY^ (*n* = 20).

### 2.4. Statistical Analysis

All values are expressed as mean ± SEM. Statistical analysis was performed by Mann-Whitney *U* test. Survival curves were analyzed with the log-rank test. Statistical tests were performed using PRISM software (Graphpad Prism). *P* < 0.05 was considered to indicate a statistical significance.

## 3. Results

### 3.1. Phenotypes of Heterozygous C3H-11^NSY^ Mice (Study 1)

**Figure 1:**
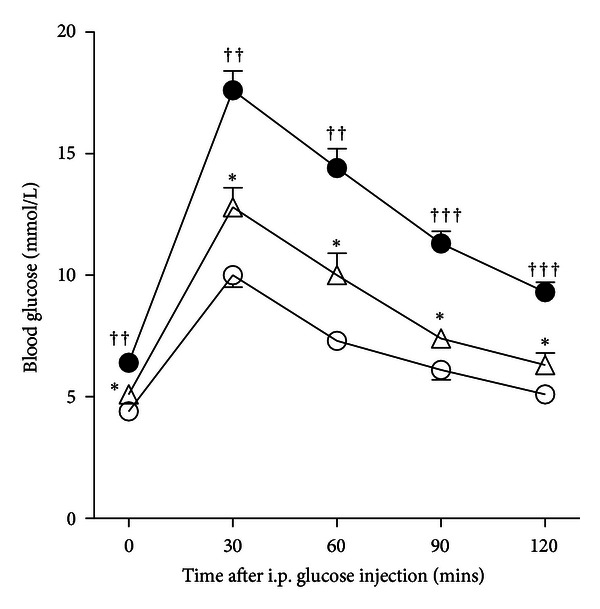
Intraperitoneal glucose tolerance test at 48 weeks of age in heterozygous C3H-11^NSY^ (*n* = 8, white triangles), C3H mice (*n* = 8; white circles), and homozygous C3H-11^NSY^ (*n* = 28, black circles, [6]). Values are mean ± SEM. *P < 0.05 compared with C3H mice (Mann-Whitney *U* test). ^††^ *P* < 0.01 and ^†††^ *P* < 0.001 compared with heterozygous C3H-11^NSY^ (Mann-Whitney *U* test).

Heterozygous C3H-11^NSY^ mice showed significantly higher blood glucose levels after fasting (*P* < 0.05) and at all time points after a glucose challenge (*P* < 0.05) than those in C3H mice (Figure 1), indicating that the introduction of a single dose of NSY-Chr11 converted normoglycemic C3H mice to hyperglycemic mice. Compared to homozygous C3H-11^NSY^ mice [6], however, heterozygous C3H-11^NSY^ mice had lower blood glucose levels after fasting (*P* < 0.01) and at all time points after a glucose challenge (*P* < 0.01) (Figure 1), suggesting that NSY-Chr11 influences glucose tolerance in an additive manner.

As shown in Table 1, insulin secretion in response to glucose as assessed by insulinogenic index tended to be lower in heterozygous C3H-11^NSY^ than in C3H mice and was similar to that in homozygous C3H-11^NSY^ mice. Fasting insulin level (0 min in Table 1) and HOMA-IR in heterozygous C3H-11^NSY^ tended to be higher than those in C3H mice.

In the anatomical analysis, body weight in heterozygous C3H-11^NSY^ mice was not significantly different from that in C3H mice (Table 2). Fat pad weight and percent fat pad weight/body weight were not significantly different either. Compared to that in homozygous C3H-11^NSY^ mice in our previous study [6], body weight was not significantly different. Fat pad weight and percent fat pad weight/body weight were not significantly different either. Those in heterozygous C3H-11^NSY^ mice, however, tended to be lower than those in homozygous C3H-11^NSY^ mice and similar to those in C3H mice.

### 3.2. Streptozotocin Sensitivity in Heterozygous C3H-11^NSY^ (Study 2)

**Figure 2:**
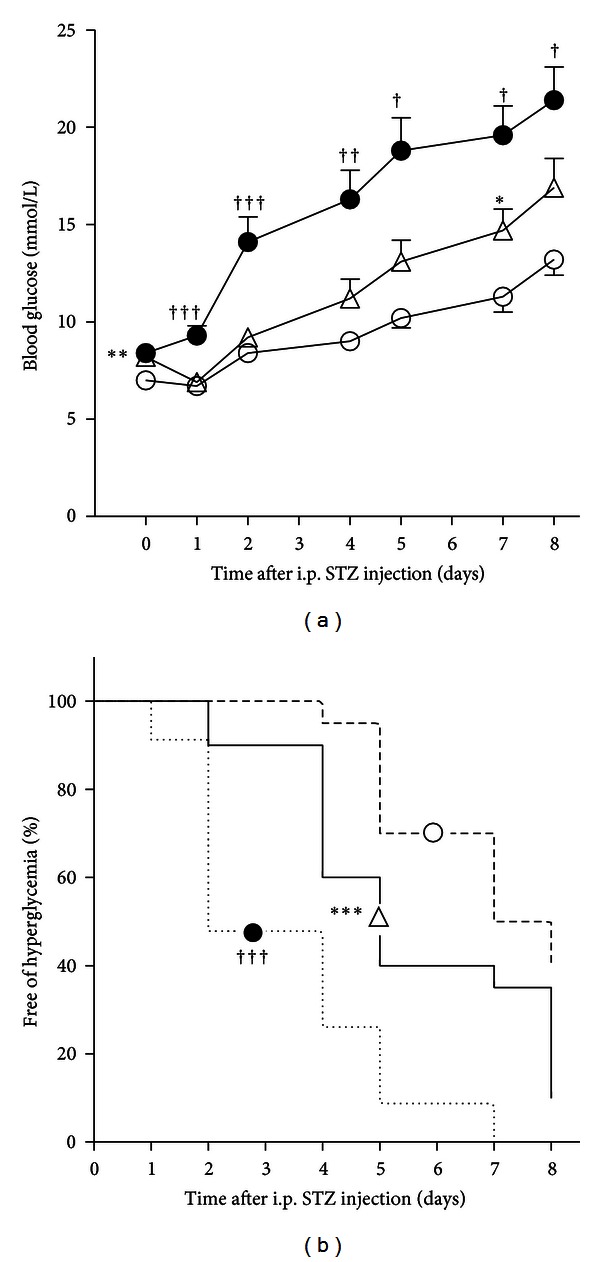
Streptozotocin sensitivity at 12 weeks of age in heterozygous C3H-11^NSY^ (*n* = 20, white triangles), C3H mice (*n* = 20; white circles), and homozygous C3H-11^NSY^ (*n* = 23, black circles, [7]). The glucose concentration was determined 0, 1, 2, 4, 5, 7, and 8 days after a single intraperitoneal injection of streptozotocin at 175 mg/kg body weight. Blood glucose concentrations are presented as mean ± SEM (a). The percentage of animals free of hyperglycemia (blood glucose level >11.1 mmol/L) is shown (b). *P < 0.05, **P < 0.01, and ***P < 0.001 compared with C3H and ^†^ *P* < 0.05, ^††^ *P* < 0.01, and ^†††^ *P* < 0.001 compared with heterozygous C3H-11^NSY^ (Mann-Whitney *U* test for (a), log-rank test for (b)).

Heterozygous C3H-11^NSY^ mice showed a higher blood glucose level after streptozotocin injection than C3H mice (Figure 2(a)), and life table analysis demonstrated a significant difference in survival curves between C3H-11^NSY^ and C3H mice (*P* < 0.001) (Figure 2(b)), indicating that the introgression of a single dose of Chr11 from STZ sensitive NSY mice converted STZ resistant C3H mice to STZ sensitive. Compared with homozygous C3H-11^NSY^ mice, however, heterozygous C3H-11^NSY^ mice showed significantly lower blood glucose levels (Figure 2(a)), and life table analysis demonstrated a significant difference in survival curves between homozygous and heterozygous C3H-11^NSY^ mice (*P* < 0.001) (Figure 2(b)), suggesting that NSY-Chr11 influences STZ sensitivity in an additive manner.

## 4. Discussion

This study clearly demonstrated that the introgression of Chr11 of the diabetes-susceptible NSY strain onto the diabetes-resistant C3H strain caused marked changes in glucose tolerance and STZ susceptibility, even in a heterozygous state. Blood glucose levels of heterozygous C3H-11^NSY^ mice were intermediate between those of homozygous C3H-11^NSY^ and C3H mice, suggesting that the mode of inheritance of glucose intolerance is additive. 

Our previous studies with reciprocal F1 crosses of NSY and C3H mice showed a different mode of inheritance depending on the phenotypes studied [8]. Hyperglycemia and fasting plasma insulin showed autosomal dominant inheritance, while impaired insulin secretion and epididymal fat accumulation showed an autosomal recessive mode of inheritance [8]. Since F1 mice are heterozygous for all autosomes, the mode of inheritance observed in our previous study with F1 mice is the combined effect of all chromosomes responsible for each phenotype. In contrast, heterozygous C3H-11^NSY^ mice in the present study were heterozygous for only Chr11, while all other chromosomes were homozygous for control C3H mice, making it possible to clarify the dose effect and mode of inheritance of Chr11 independent of other chromosomes. The data in the present study indicated an additive mode of inheritance for hyperglycemia, which is different from the dominant inheritance of hyperglycemia observed in F1 mice [8]. The differences in mode of inheritance observed in this study with heterozygous C3H-11^NSY^ mice and in our previous study with F1 mice suggest a genetic interaction of Chr11 with other chromosomes. To clarify this, studies with double and triple consomic strains, in which different combinations of chromosomes from NSY mice were introgressed onto C3H background genes, are now underway.

Our previous studies with homozygous C3H-11^NSY^ mice demonstrated that introgression of two doses of Chr11 from NSY mice onto control C3H mice converted STZ resistant C3H mice to STZ sensitive [7]. STZ sensitivity in heterozygous C3H-11^NSY^ mice in the present study was intermediate between that of homozygous C3H-11^NSY^ and that of C3H mice, suggesting a dose effect of Chr11 on STZ sensitivity and an additive mode of inheritance for this phenotype.

Although the present study with C3H-11^NSY^ mice made it possible to clarify the effect of Chr11 independent of other chromosomes, the additive mode of inheritance of glucose intolerance and STZ sensitivity observed in the present study does not necessarily reflect the effect of a single gene, but rather could result from the combined effect of multiple genes on Chr11. Further studies with congenic and subcongenic strains are necessary to clarify whether or not each phenotype observed in heterozygous C3H-11^NSY^ mice is based on a single gene or the combined effect of multiple genes.

Our previous studies with homozygous C3H-11^NSY^ mice demonstrated that impaired insulin secretion in NSY mice was accounted for mostly by Chr11 and that insulin resistance, which is independent of adiposity and obesity, was accounted for partly by Chr11 [6]. In the present study, impaired insulin secretion and insulin resistance in heterozygous C3H-11^NSY^ mice were similar to those in homozygous C3H-11^NSY^ mice, although not statistically significantly different from those in C3H mice probably due to the small number of mice analyzed. In fact, when heterozygous C3H-11^NSY^ mice in the present study were compared with our previous data of C3H mice [6], insulin secretion was significantly impaired and insulin resistance was marginally stronger in heterozygous C3H-11^NSY^ mice (*P* < 0.05 and *P* = 0.07, resp.; Mann-Whitney *U* test). Further studies with a larger number of mice are necessary to clarify the underlying mechanisms of glucose intolerance in heterozygous C3H-11^NSY^ mice, in particular the contribution of impaired insulin secretion and insulin resistance to glucose intolerance. Such studies are now underway. 

Genome-wide or large-scale association studies have revealed several candidate genes for type 2 diabetes [9–13], although the orthologues of these genes are not located on mouse Chr11. In mice, however, linkages with type 2 diabetes were reported on Chr11 in several different crosses [14–17]. This suggests the possibility that human orthologues of the diabetes-susceptibility gene(s) on mouse Chr11 are acting as rare variants in humans and therefore have not been detected by genome-wide association studies. Candidate genes on mouse Chr11 are hepatocyte nuclear factor-1*β*, GLUT4, nucleoredoxin, and glucokinase [3, 5–7, 18, 19]. Human orthologues of these genes and other candidate genes on Chr11 are important candidates for genes explaining the so-called missing heritability in human type 2 diabetes. 

## 5. Conclusions

These data indicated that the introgression of Chr11 of the diabetes-susceptible NSY strain onto diabetes-resistant C3H caused marked changes in glucose tolerance and STZ susceptibility, even in heterozygous state, indicating that Chr11 harbors gene(s) responsible for hyperglycemia and STZ susceptibility, which is effective even in a single dose. Hyperglycemia and STZ sensitivity in heterozygous C3H-11^NSY^ mice, however, was not as severe as in homozygous C3H-11^NSY^ mice, suggesting that the mode of inheritance of a gene or genes on Chr11 for hyperglycemia and STZ sensitivity is additive. The difference in the dose effect and mode of inheritance for some phenotypes between the present study and our previous study with F1 mice suggests the genetic interaction between Chr11 and other chromosomes in determining the phenotypes. The consomic strains constructed in our studies will facilitate fine mapping and identification of responsible genes for diabetes-related phenotypes, as well as gene-gene and gene-environment interactions.

## Figures and Tables

**Table 1:** Insulin secretion and insulin resistance at 52 weeks of age.

		C3H (*n* = 8)	C3H-11^NSY^ (heterozygotes) (*n* = 8)	C3H-11^NSY^ (homozygotes)^†^ (*n* = 26)
Blood glucose				
0 min	(mmol/L)	5.6 ± 0.3	5.8 ± 0.4	6.3 ± 0.3
15 min	(mmol/L)	17.3 ± 0.6	20.7 ± 2.9	18.1 ± 0.5
30 min	(mmol/L)	19.7 ± 1.9	23.4 ± 2.8	22.8 ± 0.5
ΣΔgAUC	(pmol/L × min)	281.7 ± 19.6	356.1 ± 57.7	300.2 ± 9.3
Insulin				
0 min	(pmol/L)	19.6 ± 5.3	31.7 ± 7.6	25.7 ± 2.7
15 min	(pmol/L)	140.1 ± 16.9	112.7 ± 12.9	106.1 ± 8.4
30 min	(pmol/L)	78.7 ± 8.1	88.1 ± 10.7	84.8 ± 5.7
ΣΔiAUC	(pmol/L × min)	2249.8 ± 252.6	1639.8 ± 324.4	1649.0 ± 140.3
Insulin secretion^††^		8.6 ± 1.5	5.8 ± 1.5	5.6 ± 0.5
Insulin sensitivity^†††^		118.7 ± 39.9	201.2 ± 56.9	168.4 ± 20.9

Values are total number or mean ± SEM. Heterozygous C3H-11^NSY^ were compared with C3H and homozygous C3H-11^NSY^ by Mann-Whitney *U* test, but no significant difference was observed. ^†^Data of homozygous C3H-11^NSY^ mice were already reported [6]. ^††^Assessed by insulinogenic index (incremental AUC (ΣΔiAUC), (pmol/L) divided by incremental glucose AUC (ΣΔgAUC), (mmol/L)) during ipGTT. ^†††^Assessed by HOMA-IR: calculated from basal insulin and glucose concentrations (fasting glucose (mmol/L) × fasting insulin (pmol/L)).

**Table 2:** Anatomical analysis at 54 weeks of age.

		C3H (*n* = 8)	C3H-11^NSY^ (Heterozygous) (*n* = 8)	C3H-11^NSY^ (Homozygous)^†^ (*n* = 26)
Blood glucose (ad lib)	(mmol/L)	6.7 ± 0.4	7.4 ± 0.5	8.1 ± 0.2
Insulin (ad lib)	(pmol/L)	135.9 ± 12.3	172.4 ± 16.5	226.0 ± 12.8
Body weight	(g)	31.4 ± 0.8	29.6 ± 0.8	31.0 ± 0.5
Liver	(g)	1.549 ± 0.100	1.529 ± 0.045	1.409 ± 0.039
Total fat	(g)	1.065 ± 0.109	1.024 ± 0.070	1.209 ± 0.072
Epididymal fat	(g)	0.522 ± 0.068	0.527 ± 0.042	0.707 ± 0.054
Mesenteric fat	(g)	0.543 ± 0.065	0.497 ± 0.035	0.502 ± 0.021
Total fat/body weight	(%)	3.37 ± 0.29	3.44 ± 0.18	3.87 ± 0.19

Values are total number or mean ± SEM. Heterozygous C3H-11^NSY^ were compared with C3H and homozygous C3H-11^NSY^ by Mann-Whitney *U* test, but no significant difference was observed. ^†^Data of homozygous C3H-11^NSY^ mice were already reported [6].
